# 128. Development of an Analytics Dashboard to Monitor Antimicrobial Selection and Duration for Pneumonia

**DOI:** 10.1093/ofid/ofab466.330

**Published:** 2021-12-04

**Authors:** Ashley Marx, Sydney E Browder, Jason C Liu, Michael J Swartwood, Nikolaos Mavrogiorgos

**Affiliations:** 1 UNC Medical Center, Durham, NC; 2 UNC Gillings School of Public Health, Durham, North Carolina; 3 University of North Carolina at Chapel Hill, Chapel Hill, North Carolina; 4 University of North Carolina School of Medicine Division of Infectious Diseases, Chapel Hill, North Carolina

## Abstract

**Background:**

Analytical and visual tools can be used to monitor progress for a variety of ASP key performance indicators, but few data describe the process of building disease-state specific tools to retrospectively monitor antimicrobial choice and duration. We describe process and methods for development of a pneumonia dashboard.

**Methods:**

In late 2019, the Carolina ASP began construction of a dashboard to monitor antimicrobial selection and duration in patients admitted with a diagnosis code (ICD-10) consistent with pneumonia. Data extracted from the medical record after discharge included: admission date and time, admission and discharge ICD-10s, inpatient orders and administrations for agents included in the NHSN Antimicrobial Use (AU) option, and antimicrobials ordered at discharge with associated ICD-10. Extracted data fields were validated using a one-month sample. Displays were constructed to trend selection during the first 48 hours of admission, inpatient days of therapy, and total length of therapy (sum of inpatient + outpatient days) for patients who received a discharge prescription for an antimicrobial included in the AU option that was associated with an ICD-10 consistent with pneumonia. Trends observed between Jan 2020 and Mar 2021 are reported.

**Results:**

341 admissions were trended. Within the first two days of admission, monthly proportions of patients receiving an antimicrobial by category were: anti-MRSA therapies (vancomycin, linezolid), 0.20 to 0.75; broad spectrum beta-lactams (e.g., cefepime, pip/tazo), 0.40 to 0.81; CAP therapies (e.g., ceftriaxone, levofloxacin), 0.48 to 1.00 (Figure). Median inpatient duration of therapy was 5 days (IQR 3-8; range 1 to 68). Total length of therapy was median 6 days (IQR 4-10; range 1 to 68).

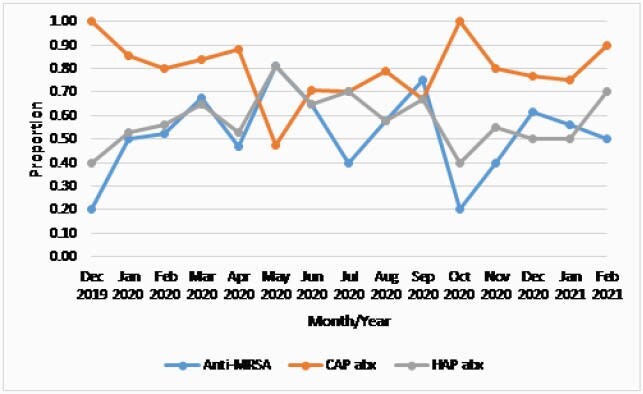

Figure. Proportions of Patients Prescribed Antimicrobial Categories of Interest During the First 48 Hours of Admissions for Pneumonia. Legend: Anti-MRSA = vancomycin or linezolid; HAP abx = cefepime, piperacillin/tazobactam, ceftazidime, meropenem; CAP = ceftriaxone, azithromycin, ampicillin/sulbactam, amoxicillin/clavulanate, cefdinir, levofloxacin.

**Conclusion:**

Automated reports and visual tools can provide actionable insights for ASP practice. From this dashboard, we identified variable but high rates of anti-MRSA and broad-spectrum beta-lactam use within the first 48 hours of admission. The median inpatient and total length of therapy of 5 and 6 days, respectively, were similar to guideline-recommended durations. The up-front cost for building analytical tools can be substantial, but can be viewed as an investment if the metrics and methods are carefully selected.

**Disclosures:**

**All Authors**: No reported disclosures

